# Suitability of Inexpensive Eye-Tracking Device for User Experience Evaluations

**DOI:** 10.3390/s18061822

**Published:** 2018-06-05

**Authors:** Gregor Burger, Jože Guna, Matevž Pogačnik

**Affiliations:** Faculty of Electrical Engineering, University of Ljubljana, 1000 Ljubljana, Slovenia; joze.guna@fe.uni-lj.si (J.G.); matevz.pogacnik@fe.uni-lj.si (M.P.)

**Keywords:** user experience, eye tracking, DriveGreen, user study, user interface, the Eye Tribe, Ogama

## Abstract

We present the results of a study evaluating the suitability of an inexpensive eye-tracking device for the enhancement of user experience evaluations. Ensuring a comfortable user experience is an important part of the mobile application design process. Evaluation of user experience is usually done through questionnaires and interviews, but it can be improved using eye tracking sensors for user experience studies. We conducted a user experience study of DriveGreen, a mobile application devoted to ecodriving for a transition to a low-carbon society. We used an inexpensive eye-tracking device in addition to standard User Experience Questionnaire and Single Ease Question questionnaires. The results show that the inexpensive eye-tracking device data correlate with data from User Experience Questionnaire and Single Ease Question questionnaires and interviews with users. We conclude that an enhancement of user experience evaluations with inexpensive eye-tracking device is possible.

## 1. Introduction

The aim of the DriveGreen project [1] was the development of a motivational eco-driving application for a transition to a low-carbon society. The project was a result of cooperation between electrical engineers and anthropologists. A motivational Android mobile application was developed, with the goal to help lower CO_2_ emissions by monitoring users’ mobility habits and encouraging eco-driving, public transport and cycling/walking. The application was named “1, 2, 3 Ljubljana” and is accessible free of charge in the Google Play app store [2]. User Centered Design (UCD) principles were followed during the application design process, with the intent to design intuitive, easy to use application suitable for on the road or vehicles uses. We used an eye-tracking sensor device to enhance the evaluation of user experience.

Sustainability [3] is an important aspect of a modern, eco-aware society. Consequently, new design challenges emerge. Gabrielli et al. [4] explored the challenges of designing and assessing motivational features for effective and long-lasting behavior change for sustainable mobility. Innovative methods of user engagement and motivation are required to promote a change of users’ behavior. Kazhamiakin el al. [5] explored the potential of gamification mechanisms to incentivize voluntary behavioral changes towards sustainable mobility solutions. This research is based on the work done within the STREETLIFE EU Project, aiming towards developing games on top of existing services and systems within a smart city.

Significant impact can be achieved with relatively simple solutions, especially if these solutions are tailored to local lifestyles and take into account habits, routines, practices, requirements and expectations of the people. The DriveGreen project [6] is presented from an ethnographical and sociological point of view. Initially, the goal of the DriveGreen project was to create a simple and free smartphone application for drivers, which would provide an alternative to applications such as the Toyota’s iPhone application “A Glass of Water”, which determines and visually communicates how economical, safe, and environmentally responsible our driving is. The research within the DriveGreen project started with studying the users’ driving habits and motivational triggers for a transition to a low-carbon society. Based upon this research, the first user interface and functionalities of the application were designed. Detailed overviews of the user interfaces, interactions, modalities and quantitative measurements of driving styles were examined in a follow-up study by Burger et al. [7]. The researchers were encouraged by the research findings showing that such approaches can actually save up to 10 percent of fuel and reduce a similar proportion of CO_2_ emissions caused by traffic, Barkenbus [8], Tulusan et al. [9]. However, rather than trying to change the way people drive, the goal was to incentivize them to not use their cars at all—or at least most of the time. With this in mind, we prepared a new concept for a smartphone application that shows users the shares of their mobility modes (walking, cycling, using public transport and driving) on a daily, weekly, monthly, and annual basis. Ecological driving per se became of secondary importance to their solution, while the application was trying to motivate users to use alternative modes of transport.

Usability and the overall user experience (UX) of an interactive mobile application may be a deciding factor for the user adoption of a solution.

To ensure good user experience, the users should be involved at all stages of the development process, which is commonly referred to as User-Centered Design (UCD) approach according to Lowdermilk [10]. Choosing the right methodology and measuring the user experience Albert et al. [11], Sauro et al. [12], however, is not a trivial task. It depends on the product or service itself and is also influenced by the target user group. In Vermeeren [13], different user experience evaluation methods are presented and analyzed based on the product development phase and the studied period of experience criteria. The analysis reveals development needs for UX evaluation methods, such as early-stage methods, methods for social and collaborative UX evaluation, establishing practicability and scientific quality, and a deeper understanding of UX.

Another important aspect of user experience is the usability factor. One of the most standardized methods measuring the usability is the System Usability Scale (SUS) [14]. Instead of complex questionnaire based evaluation methods, simpler, post-task ratings of difficulty in a usability test have the potential to provide useful information and be an additional measure of user satisfaction. These can consist of as few as only one question, evaluated on numerical scales. Research in Sauro et al. [15] provides insights into a comparison of three one-question based post-task usability questionnaires. Three one-question rating types were compared in a study with 26 participants who attempted the same five tasks with two software applications. The types were a Likert scale, a Usability Magnitude Estimation (UME) judgment, and a Subjective Mental Effort Question (SMEQ). The results show all three types could distinguish between the applications with 26 participants, but the Likert and SMEQ types were more sensitive to small sample sizes.

More complex aspects of the user experience can be measured by various standardized questionnaire based methods. One of the more commonly used methods is the User Experience Questionnaire (UEQ), which is analyzed in detail in Laugwitz [16]. It consists of 26 items, measuring six factors of user experience: Attractiveness, Perspicuity, Efficiency, Dependability, Stimulation, and Novelty. The questionnaire is language dependent; however, the studies conducted for the original German questionnaire and an English version indicate a satisfactory level of reliability and construct validity.

Following the established practices of user experience and interaction design, we first performed a pilot study using a small group of participants, proposed by Nielsen [17], to evaluate the first wireframes of the application. This was followed by an extensive user experience study using standard questionnaires and the eye-tracking device. The selected participants were representative members of defined user groups and personas (based on age, gender, education and technical knowledge criteria) chosen during the design phase of the application.

Eye-tracking, Bojko [18], Mele et al. [19], and Eraslan [20], is now frequently employed to help evaluate and improve designs (from websites to product packaging) at various stages of the development cycle. It provides valuable insight into the way the users actually use devices and applications and provides objective data for further analysis. There are various methods of interpretations and visualizations of the eye-tracking data, with the heat maps (also known as attention maps) and gaze plots/scan paths (also known as gaze fixations/gaze transitions) being most commonly used. A heat map represents the values of a variable (for example, gaze duration) as colors, where the amount of “color heat” is proportional to the value of the represented variable. A gaze plot is an image showing individual’s gaze fixations represented as dots, and gaze saccades represented as lines. The diameter of a dot is proportional to the duration of the fixation, with larger dots illustrating longer fixations.

User experience testing with questionnaires and eye-tracking devices is not new, relevant tools and methods were created in the past in Albert [11]. However, the described tools and methods are expensive and sometimes difficult to use. Devices from the upper side of the price spectrum offer a broad collection of features and supported tools for data analysis. On the lower side of price spectrum, the devices offer only limited features and data analysis tools. Therefore, the open source software and development of own tools are the only alternatives. Our goal was to validate/correlate the subjective data from user experience questionnaires with the objective data from a low priced eye-tracking sensor. Through market research, we identified two eye-tracking devices: Tobii EyeX [21] and the Eye Tribe Pro [22]. Both devices work on the principle of detecting reflected IR light from peoples’ eyes, initially radiated by the eye tracker device.

Ooms [23] compared the accuracy and precision of the inexpensive Eye Tribe tracker and a professional comparable eye tracker SMI RED 250. The accuracy was measured through the distance between the recorded fixation locations and the actual location. Precision was represented by the standard deviation of these measurements. The results show that, with a correct setup and selection of software, the quality (accuracy and precision) of the recorded data can be considered comparable to that obtained by a significantly more expensive device. In Dalmaijer [22], the Eye Tribe tracker is compared to the professional EyeLink 1000 tracker. The authors showed that the spatial precision and accuracy of the inexpensive Eye Tribe tracker are good enough for fixation checking, point-of-regard analyses, and pupilometry. However, the low sampling rate renders the device unsuitable for testing high-accuracy saccade metrics. The Tobii EyeX tracker is analyzed in Gibaldi et al. [21], where the observed performance of the Tobii EyeX (i.e., accuracy < 0.6°, precision < 0.25°, latency < 50 ms and sampling frequency ≈ 55 Hz), indicates its suitability for measuring fixation parameters, saccadic, smooth pursuit and vergence eye movements. Again, the relatively low sampling rate and moderate precision limit the suitability of the Tobii EyeX for monitoring micro-saccadic eye movements or for real-time gaze-contingent stimulus control. These results indicate that inexpensive eye tracking devices can be used in user experience research and can provide valuable objective data.

The key contribution of this paper is to show an added value of inexpensive eye tracking device in user experience studies. 

The rest of the paper is organized as follows: following the Introduction, the methodology and a detailed experiment setup and procedure are described. These are followed by the results and discussion. Key conclusions and future work references are drawn in the final section.

## 2. Materials and Methods

This section describes the study with an emphasis on the procedures, participants and evaluation metrics used in the experiment. The description includes the subjective and objective variables and explains the limitations of the apparatus used. The main research questions in this study were:

- Are inexpensive eye tracking devices a suitable accessory for objective user experience evaluations?
- What is the user experience of the “1,2,3 Ljubljana” mobile application?

### 2.1. Participants

We recruited 32 participants for the experiment, of which 16 were female and 16 were male participants. The average age was 30.9 years (SD = 10.8 years), with the youngest participant being 20 years old and the oldest 61 years old. All participants were mobile phone users with mixed technical background. Sixteen of them declared themselves as advanced mobile phone users with deep technical knowledge about the phone and mobile applications. Eleven of them used phones and mobile applications on a daily basis and five used only basic mobile phone functionalities such as phone calls and short messaging. Twenty-one participants were Android phone users, eight were iPhone users and three were Windows Phone users. The majority of them reported one to two hours of phone usage per day (15 participants), some of them used the phone for two to four hours per day (9 participants) and a minority (8 participants) reported four to seven hours of phone usage per day. The most used applications among the participants were news reading applications (23 participants), weather applications (21 participants), educational applications (20 participants), entertainment and music applications (17 participants) and health and fitness applications (12 participants).

Since the inclusion of the eye-tracking device was an important part of the user experience study, we were also interested in participants’ sight. Twelve participants used distance spectacles and/or contact lenses, with all of them reporting no problems seeing the information on the mobile tablet with the sight correction accessories. We also conducted the Ishihara color perception test [24] and found only five cases of slight color deficiency. Two participants had a mild green color deficiency while another one a mild red-green color deficiency. The frequency of the color deficiency among the participants was as expected, Simunovic [25].

### 2.2. Apparatus

**Figure 1:**
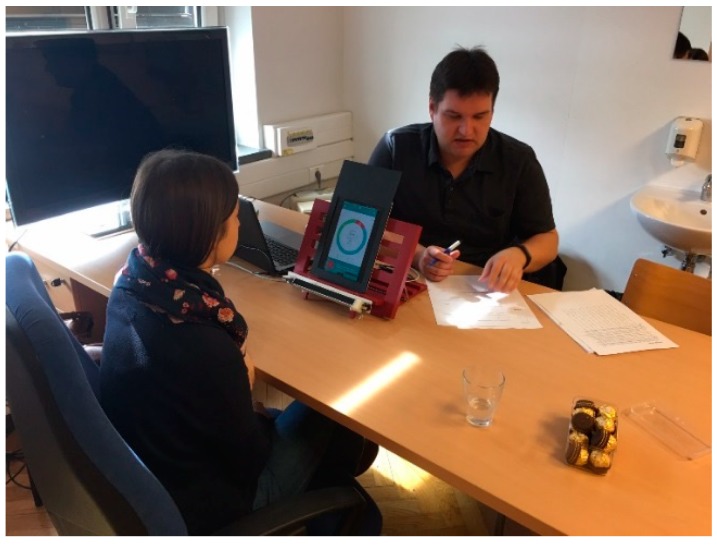
Experiment test setup.

As the eye tracking device used in the experiment is not supported on the Android system, we used a workaround, which would show the user interfaces on the mobile device and support the eye tracking process at the same time. Therefore, we used a mid-range multimedia notebook (Asus X550LB notebook with i7 CPU, 8 GB DDR 3 1600 MHz, Crucial MX100 SSD, Nvidia 740 M GPU). The notebook was used for displaying the user interfaces on an iPad 3 tablet as a second screen, using the Splashtop Wired XDisplay—Extend & Mirror application. At the same time the notebook was running the eye tracking software tool Ogama version 5.0, supported by the Eye Tribe Pro eye tracking device. The eye tracking device and the tablet were placed on a modified bookstand and a 3D printed bracket was used to secure the eye tracker to the bookstand, presented in Figure 1.

The user interface was built from a series of interactive webpages, loaded onto a web server running on the notebook and displayed on the mobile tablet. Interactive web pages were used to overcome the limitation of unsupported web page formats in the eye tracking software tool Ogama and missing functionality of live view on the eye tracking device. A virtual server with MS IIS functionalities running on VMW platform was used.

The Eye Tribe Pro tracker is a low priced device costing $199. A low price combined with the availability and free access of C# and C++ programing libraries and a less restrictive user license made the eye tracker the preferred choice. The capabilities of the Eye Tribe Pro eye tracking device are presented in Table 1.

The manufacturer supplied analysis tools were in the beta phase and not up to the standard needed to perform the user experience study with thirty-two users. Therefore, we used the open source tool Ogama (Open Gaze and Mouse Analyzer) [26] version 5.0 for eye tracking data collection and analysis. Ogama allows for recording, importing and analyzing of eye- and mouse-tracking data in parallel. It contains tools for stimuli design (application screens in our case), data recording and data analysis provided by additional sub tools and is released under GNU General Public License (GPL) Version 3. The experiment was made using the following settings: the gaze sampling rate was 60 Hz, maximum distance in pixels, which can be considered the same gaze fixation position, was 20 pixels and the fixation time in milliseconds above 2 ms correlates with the gaze fixation circle diameter in pixels.

### 2.3. Metrics

As the dependent variables in the experiment, we measured the user experience. The independent variables were the measured features of stimuli. The controlled variables included the environment in terms of using the same room and light conditions, same furniture, and equipment.

As a subjective user experience measure the User Experience Questionnaire [16] and Single Ease Question questionnaire [15] were used. User Experience Questionnaire is composed of twenty-six statements rated through the 7-stage Likert scale. The user rated statement values are calculated into six scales: attractiveness, perspicuity, efficiency, dependability, stimulation, and novelty. Higher scores indicate greater levels of agreement with the scales, while lower scores indicate greater levels of disagreement. The scores are presented on the scale ranging from −3 to +3, all scores above 1 value are considered as positive evaluation. Single Ease Question was used to evaluate the user experience in a supplementary way, as participants quantized the difficulty of completing the scenarios using 7-stage Likert scale. Value 1 on the scale meant a “Very difficult” scenario, while value 7 meant a “Very easy” scenario to fulfil.

As an objective measurement, the gaze and fixations were collected and the raw physiological data were processed using the Ogama tool. Additional statistical analysis was performed. Raw data were pre-processed (outlier and moving artefacts removed). The data processing included calculation of attention maps, gaze fixations with gaze transitions and average time to complete the scenario. The attention maps are often referred to as heat maps and present the aggregated amount of users’ fixation to a particular part of the screen. The extent of the colored area in a heat map depends on the heat map settings, and it is quite possible that an area not marked with a color actually did receive some, but limited fixations [18]. They represent only spatial information about user fixations.

Gaze fixations and gaze transitions are images representing individual’s fixations as dots and saccades between them as lines. They are also referred to as gaze plots/scan paths [18]. The size of the dots is proportional to the duration of the fixation, with larger dots illustrating longer fixations. As the dots are numbered, they represent both spatial and temporal information.

Finally, the areas of interest (AOI) were defined for different scenarios, allowing for a statistical analysis of gaze fixation durations in target AOIs.

**Figure 2:**
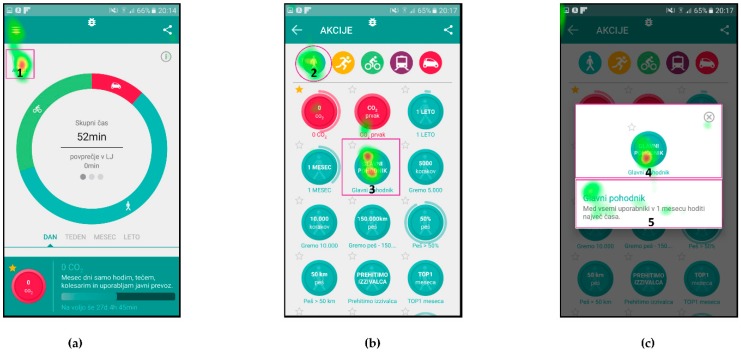
Areas of Interest defined for Scenario n. 9: (**a**) home screen of the application; (**b**) screen for listing all the available actions “Akcije” for the user; and (**c**) selected action description.

The AOIs for different scenarios were defined on individual user interface screens and represent the target areas, which should receive most of the gaze fixations, depending on the given scenario. They are presented as purple shapes (see Figure 2), such as circles in top left corner and center of Figure 2b or a square in top left corner of Figure 2a. This methodology allows for extraction of gaze fixation durations for target AOIs and consequently a quantitative analysis of the user interface experience. The latter is done through the calculations of the percentage of gaze fixation durations in target AOIs for individual user interface screens. To do that, one must also define the entire screen as an AOI.

The main goal of this analysis was to find out whether the correlation exists between the users’ experience, indicated by the UEQ and Single Ease Question SEQ scores and objective parameters, which were measured. While the UEQ score is a general and aggregated score for all users and scenarios, the SEQ score gives insight into individual scenarios. We were looking into correlations between UEQ scores, the attention maps, SEQ scores given to the scenario and AOIs. Specifically, we were looking into the correlation between the percentage of gaze fixations received by the target AOIs in a scenario, the attention maps and corresponding SEQ scores. Additionally, we compared the gaze fixations and the time needed for scenario completion.

Our assumption was that, in the case of good user experience, the eye tracking data would show the areas of users’ viewing focus on the “target” areas of the user interface for a given scenario. Furthermore, the assumption was that the duration and location of gaze fixations for individual users would correlate with the time needed to complete the scenario.

### 2.4. Experiment Environment 

The experiment was conducted in an office at the faculty premises. We put a significant emphasis on the controlled environment conditions. Air temperature, relative humidity and lighting conditions were controlled. The tests were conducted using the same testing equipment, including the chair.

### 2.5. Tasks and Procedure

We conducted the experiment with one participant at a time. One researcher was conducting the experiment while the other was observing the participant and noting the participant’s explicit and implicit responses, such as comments, questions, body language, facial expressions, etc.

The participation was on a voluntary basis. Prior to the experiment, the participants were informed about the general instructions on how to participate in the study. Experiment was divided into three parts. In the first part, the participants were welcomed and the procedure and aims of the experiment were explained. In the next step, the consent was signed and the basic demographic and technology background information was collected. The collection of participants’ sight information (use of spectacles, contact lenses, and Ishihara color perception test) followed to ensure only healthy volunteers were selected.

The second part the study was conducted in a balanced manner. The participants were presented with scenarios on a random, preselected order [18]. Immediately after completing the given scenario, the SEQ was used to determine the difficulty of the scenario. Scenarios were rounded-up with the UEQ questionnaire to determine the user experience.

We prepared thirteen scenarios for testing of mobile application’s user experience. The scenarios covered the tasks like finding the mobile application’s icon and launching the application, checking of user’s personal achievements, working with the side menus, selecting user’s motivational challenges or modifying the application settings. The difficulty varied in steps needed to complete the scenarios, but did not exceed more than five steps or two minutes in time to complete the task. During the user study, we used the counterbalancing technique for the succession of the scenarios as suggested in the literature by Williams [27]. Short scenario descriptions are presented in Table 2.

In the third part, after the experiment, a short follow-up interview was performed and participants’ feedback was collected. Participants quantized the responses of follow-up statements with SEQ questionnaire on a 7-stage Likert scale. Value 1 on the scale meant “Strongly disagree” and value 7 meant “Strongly agree” with the statement. The duration of the experiment was approximately 1 h per participant.

## 3. Results

This section presents a set of results which are discussed in the next section. The goal of this experiment was to estimate the suitability of an inexpensive eye tracking device as an enhancement of user experience evaluations. Before the experiment, we set the hypothesis that the eye tracking data will correlate with the user experience results and give us additional confirmation or even an explanation of the latter’s numerical values. Therefore, the eye tracking data were compared to two sets of user experience objective parameters: the UEQ parameters and the task difficulty, given by the users through the SEQ questionnaire. As described in previous sections, three information parameter sets were obtained through the eye tracking device: (1) the attention maps; (2) the gaze fixations with gaze transitions; and (3) the duration of gaze fixations in defined areas of interest.

**Figure 3:**
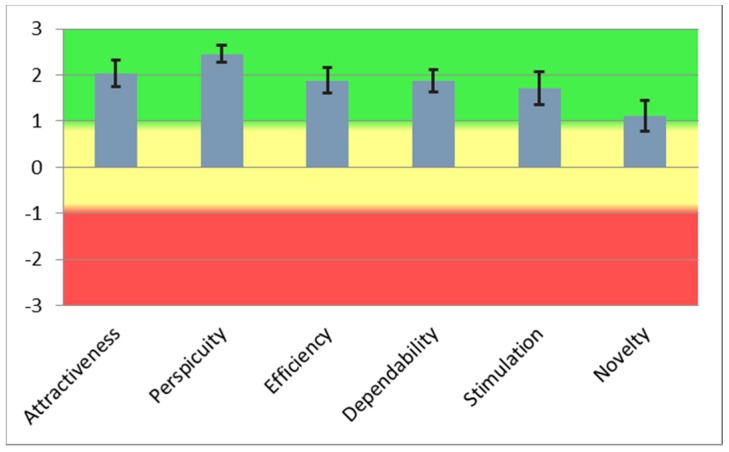
UEQ score for the Drive Green application.

The aggregated user experience results are presented in Figure 3, where aggregated UEQ values indicate an above average satisfaction with the application. For comparison with the eye tracking data, the most suitable UEQ values are UEQ perspicuity and UEQ efficiency, as they indicate the intuitiveness of the user interface.

**Figure 4:**
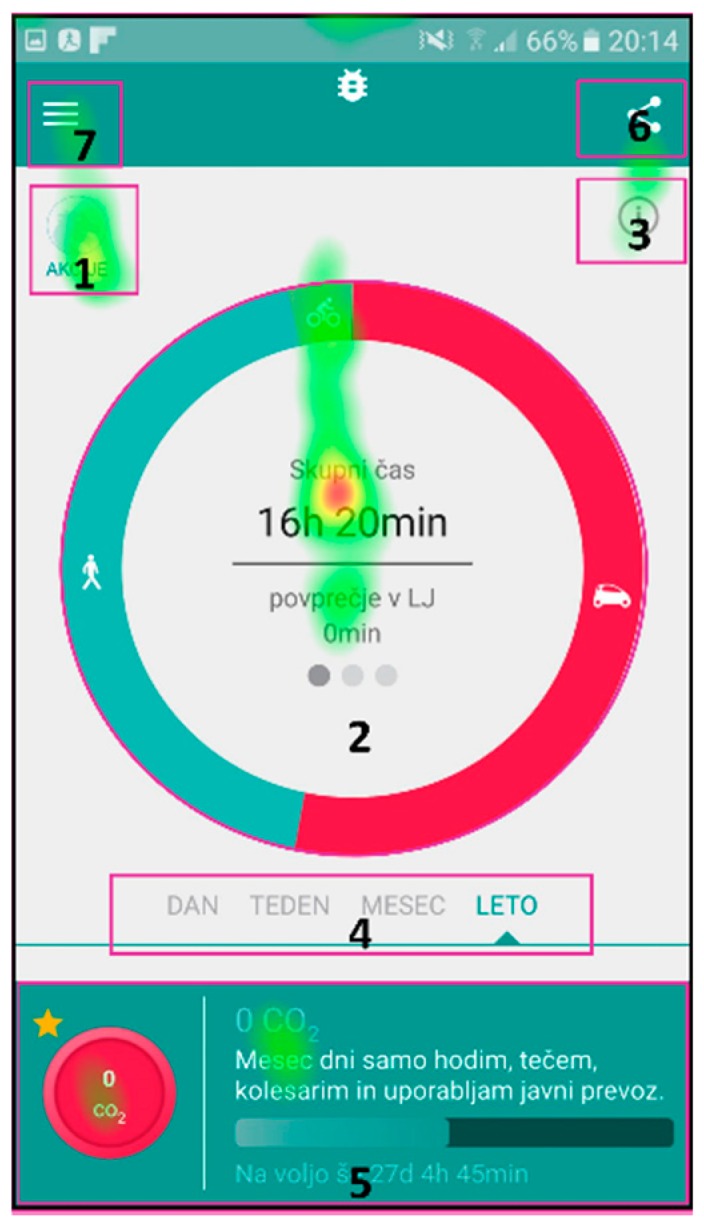
Attention map for Scenario n. 3.

**Figure 5:**
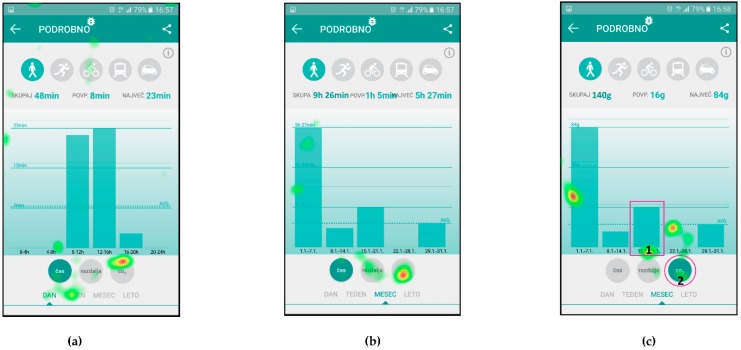
Attention maps for Scenario n. 7: (**a**) time of walking in a one day; (**b**) time of walking in one month; and (**c**) CO_2_ saving because of walking in one month.

**Figure 6:**
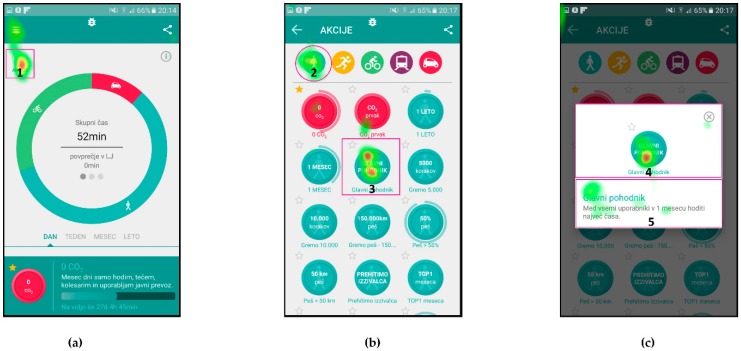
Attention maps for Scenario n. 9: (**a**) home screen of the application; (**b**) screen for listing all the available actions “Akcije” for the user; and (**c**) selected action description.

The eye tracking data, which are the most suitable for comparison with UEQ results are the attention maps. Both the UEQ and attention maps represent data gathered from all users and are aggregated. The attention maps for the three selected scenarios are presented in Figure 4, Figure 5 and Figure 6, while quantitative data regarding gaze fixations are presented in Table 3, Table 4 and Table 5.

Figure 4 presents the attention map for Scenario n. 3, where the users were given the task to describe the main functional parts of the screen with an emphasis on the user activity data.

Table 3 presents the quantitative analysis data of the gaze fixations for Scenario n. 3, aggregated for all users. 

Figure 5 presents the attention maps on three consecutive screens for Scenario n. 7, where the users were asked to check the amount of CO_2_ reduction due to their walking activities (instead of driving), in the third week of January. The users had to identify the icon for CO_2_, identify the correct week and read the CO_2_ value on the left-hand side of the screen.

Table 4 presents the quantitative analysis data of the gaze fixations for Scenario n. 7 (for Figure 5c).

Figure 6 presents the attention maps for Scenario n. 9, where the users were asked to find the action item named “Glavni pohodnik” (English: “Top walker”) and find the requirements necessary to achieve this action’s goal. In this scenario, the users had to find the icon “Akcije” (English: “actions”) on the first screen, icon of a walker and action item on the second screen and read the description of the requirements on the third screen.

Table 5 presents the quantitative analysis data of the gaze fixations for Scenario n. 9—aggregated for all users.

In addition to the attention maps, and gaze fixation durations, which show aggregated fixation data from selected subjects, the gaze fixations with gaze transitions for individual users are also presented. 

**Figure 7:**
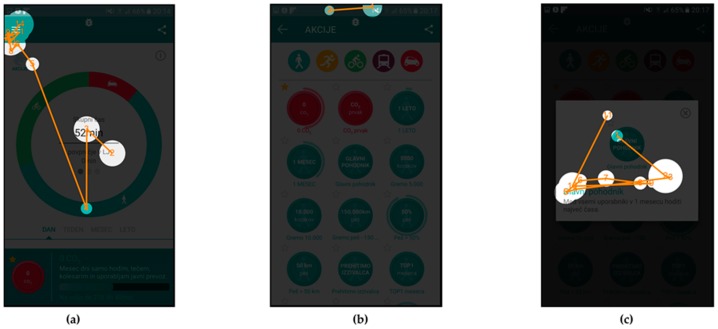
Gaze fixations and transitions for User id130 during Scenario n. 9: (**a**) home screen of the application; (**b**) screen for listing all the available actions “Akcije” for the user; and (**c**) selected action description.

**Figure 8:**
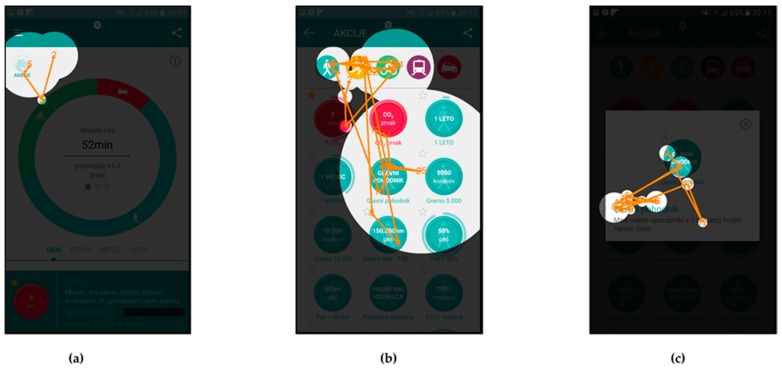
Gaze fixations and transitions for User id103 during Scenario n. 9: (**a**) home screen of the application; (**b**) screen for listing all the available actions “Akcije” for the user; and (**c**) selected action description.

**Figure 9:**
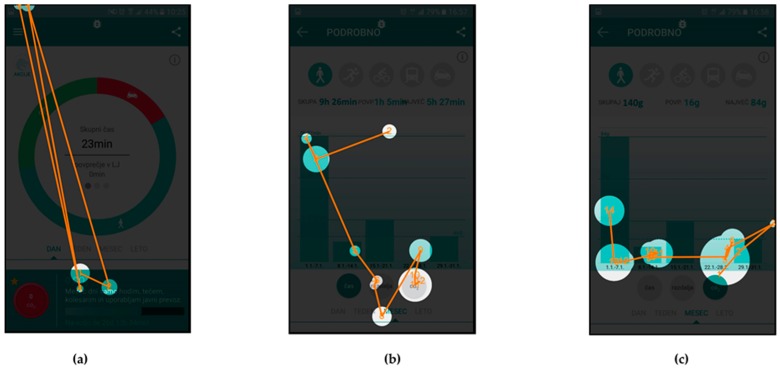
Gaze fixations and transitions for User id130 during Scenario n. 7: (**a**) home screen of the application; (**b**) time of walking in one month; and (**c**) CO_2_ saving because of walking in one month.

**Figure 10:**
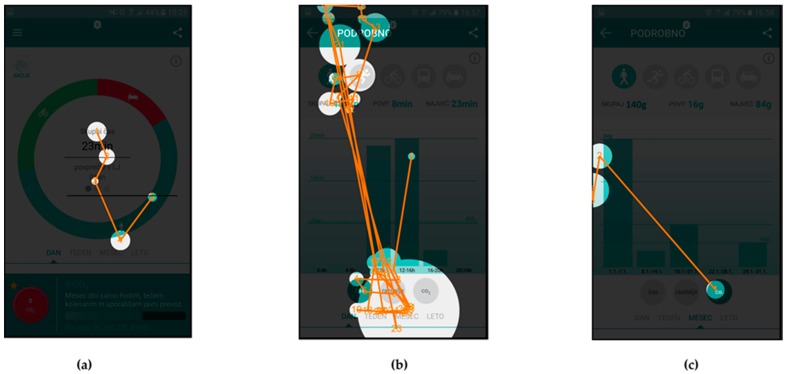
Gaze fixations and transitions for User id103 during Scenario n. 7: (**a**) home screen of the application; (**b**) time of walking in one month; and (**c**) CO_2_ saving because of walking in one month.

Fixations and transitions for Scenario n. 9 are presented in Figure 7 (User id130) and Figure 8 (User id103), while gaze fixations and transitions for Scenario n. 7 are presented in Figure 9 (User id130) and Figure 10 (User id103). 

Finally, the UEQ and the SEQ results are presented in Table 6. Users id103 and id130 were chosen as examples because User id103 was among slower users in performing the scenario tasks and rated the application with lower UEQ scores (1.5 for perspicuity and 1.25 for efficiency), while User id130 was among the faster ones and gave higher UEQ scores (2.75 for perspicuity and 2.25 for efficiency). As such, they represent different types of users and seemed suitable for demonstration of the results. Additionally, these two users also rated the scenario difficulties differently, with User id 103 having one of the lowest SEQ average values for all scenarios and User id130 having one of the highest values. This also correlates with other data as a lower SEQ value represents higher difficulty.

**Figure 11:**
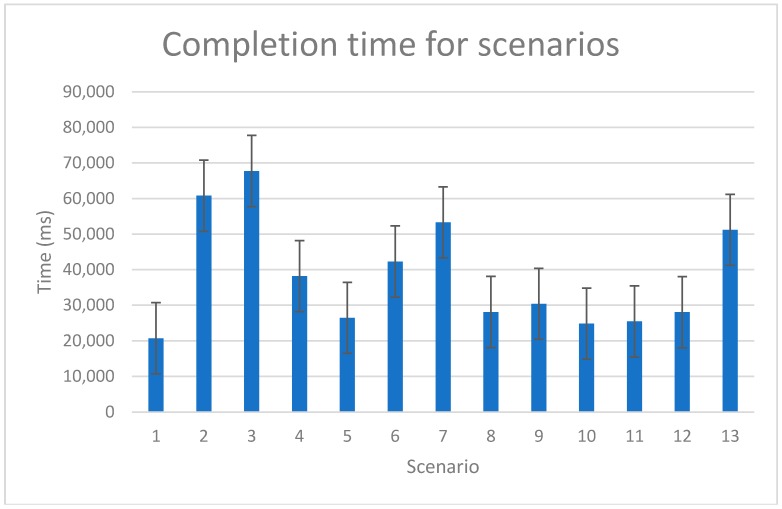
Completion time for scenarios.

Figure 11 presents the average time (ms) it took users to successfully complete the individual scenario, including the standard deviation. Scenarios n. 2 and n. 3 took users the most time to complete. In these scenarios, the users had to identify and name the application features that they recognized on the application’s user interface, once using the built-in help feature and the second time without it. Scenario n. 4 asked of users to find the aggregated data for the current day including the distance covered, activity duration and CO_2_ savings. Scenarios n. 6 and n. 7 required of users to check the amount of CO_2_ reduction and walking time in the third week of January. In the last scenario, Scenario n. 13, the users had to find their position on the leader board.

Table 7 lists the times needed for completion of Scenarios n. 6, n. 7 and n. 9 for Users id103 and id130. User id103 first completed Scenario n. 6 than Scenario n. 7 and finally Scenario n. 9. User id130 first completed Scenario n. 9, then Scenario n. 7 and finally Scenario n. 6. Scenarios n. 6 and n. 7 are similar in design. Scenario n. 6 asks the user to find the data for distance traveled in a current day, while Scenario n. 7 asks the user to check the amount of CO_2_ reduction and time of walking activities, in the third week of January. Steps needed to complete the task are similar; the only difference is in the final steps where the user selects different time scale and activity. The time results show reduction of time needed to complete the task when user is performing a task, similar to the one that he/she successfully completed before. The data for User id103 and User id130 confirm this. Overall, User id130 completed the tasks in less time than User id103.

SEQ scores for Scenarios n. 4, n. 6, n. 7 and n. 13 are noticeably lower than the rest of the SEQ scores. The lower SEQ scores also correlate with the completion time for these scenarios—a lower score represents a more difficult scenario and longer completion time. Exception are Scenarios n. 2 and n. 3, which got higher SEQ scores even though it took users the most time to complete them. We can attribute this to the extensive demands of Scenarios n. 2 and n. 3.

Most of the given scores were in region of value of 5, 6, or 7 on the scale of 1–7. No value lover that 3 was given by the users.

## 4. Discussion

The users successfully completed all given scenarios, and none of the users reported the scenario as too difficult to complete. In a few cases, we detected missing or incomplete eye tracking data because individual users moved during the experiment or accidentally covered the eye tracker with hand while completing the experiment. 

In this section, the general UEQ and SEQ related discussion is presented, followed by the discussion regarding the correlation with the eye tracking data.

The aggregated UEQ questionnaire results had a positive value of 1 or more for all six UEQ scales (see Figure 3). The highest UEQ score was given to Perspicuity scale, which obtained a value of 2.45. Perspicuity is calculated based on selected word pairs such as not understandable–understandable, easy to learn–difficult to learn, complicated–easy, and clear–confusing. With these phrases, we basically describe good user experience. The UEQ Novelty scale provides an insight into how new/novel and attractive the application is. Although the result is positive, the value of 1.08 is not on the level of the UEQ Perspicuity scale. This is probably because similar mobile applications already exist on the market, and the users are familiar with this fact, even though these applications are not adapted to the local environment as the DriveGreen application is.

With the SEQ questionnaire, we measured the difficulty level of the scenarios. As we have assumed, the results gave high scores for application’s intuitiveness and ease of use. Users did report some problems with navigation and understanding of the user interface, but we detected a noticeable decrease in problems and a higher-level understanding of the user interface, after the users have completed a similar scenario beforehand. 

Based on the SEQ results, the study observation and measured scenario completion time, it is obvious that some scenarios were more difficult to complete than the others (see Table 8). Scenarios n. 2 and n. 3 took the users the most time to complete as they required users to describe the functionalities of the application based on what they saw on the screen. In Scenario n. 2, users first had to find the “help option”, which provided written instructions of the application features. Scenario n. 3 had the same instruction as Scenario n. 2, but without using the “help option”. Surprisingly, the scenario completion times do not differ a lot, which is an indication of a reasonably clear and intuitive user interface. 

Scenario n. 6 required users to find the data about the distance traveled in the current day and Scenario n. 7 required users to check the amount of CO_2_ reduction and time of walking activities, in the third week of January. These two scenarios, in addition to Scenario n. 13, were reported as the most difficult to complete by the users as they had the lowest SEQ score. The biggest user complaint regarding the intuitiveness of the application was a lack of indication that the elements of the activities circle (Figure 2) were clickable. Scenarios n. 6 and n. 7 were expected to be the hardest to complete and the measured data correlate with expectations. In Scenario n. 13, which surprisingly also took users above average time to complete, users had to identify their place on the leader board for running activity. Most users found the leaderboard with no or little help. When the leader board was selected, it showed user data for walking and users had to switch to running. Most users reported forgetting the final step, which consequently prolonged the time for completion of the scenario.

The eye tracking results, which are most suitable for comparison with the UEQ results, are the attention maps. Attention maps presented in Figure 4, Figure 5 and Figure 6 provide aggregated eye tracking data for all the users together in the experiment for selected scenarios. The colored parts of the user interface represent those parts to which the users payed most attention to. One can identify a matching correlation between the parts of the screen the users paid attention to, and the users’ comments and experiment observations, which correlates with good user experience results of the UEQ questionnaire.

The quantitative analysis is based on the gaze fixation duration data for AOIs presented in Table 3, Table 4 and Table 5, which indicate a correlation with the SEQ results, as described below. The results are also commented taking into account the attention maps information. Furthermore, the analysis is extended with an analysis of gaze fixations/transitions and time spent for selected scenarios.

Figure 4 presents the attention map for Scenario n. 3, where the users were given the task to describe the main functional parts of the screen with an emphasis on the user activity data. The users obviously payed most attention to the main functional parts of the screen with an emphasis on the user activity data.

Additionally, the data in Table 3 show that the user activity data (29%) and the selected action description (22%) captured most of the users’ attention, while other AOIs captured less. The total percentage of users’ interest in defined AOIs sums up to 62% of gaze fixation time, which correlates with relatively high SEQ score of 6.52 for Scenario n. 3 (see Table 8), which can be interpreted as a reasonable intuitiveness of the user interface, thus reducing the scenario difficulty.

Figure 5 presents the scenario where the users were asked to check the amount of CO_2_ reduction due to their walking activities, in the third week of January on three consecutive screens. Not only was the eye-tracking device able to detect the most important parts of the user interface, but it could also distinguish between the selectors for time (Slovenian: Čas), distance (Slovenian: Razdalja), CO_2_ and time line in the bottom of the user interface. It also seems that the users were checking whether the walking activity was selected as there are some attention indicators on the top of the screen around the activity icons (Figure 5a). 

The gaze fixation data for AOIs in Table 4, referring to the user interface in Figure 5c, show, that 23% of users’ attention was placed on the target AOIs, with some of it spent on the graph of CO_2_ reduction (8%) and a some more on the selector for CO_2_ icon (15%). Even though these AOIs are relatively small when compared to the entire screen, these gaze fixation percentages are lower than the ones for other two scenarios. As the SEQ score for this scenario was lowest of all three scenarios with a value of 5.13 (see Table 8), it seems there is a correlation with lower percentages of gaze fixations in the target AOIs.

Figure 6 presents the attention maps for the action item named “Glavni pohodnik” (English: “Top walker”), where the scenario asked users to and find the requirements necessary to obtain this action item. In Figure 6a it is shown that Users identified the icon “Akcije” (English: “actions”) in the top left corner, which leads them to the second screen with listed available icons (Figure 6b). Attention map again clearly shows that users’ attention was on the icon for selection of the walking activity and on the icon “Glavni pohodnik” (English: “Top walker”). Figure 6c represents the users’ attention focus during reading of the instructions for this activity. 

The gaze fixation data for Scenario n. 9 are presented in Table 5. The relatively small AOI for icon “Akcije” (English: “actions”) received 30% of users’ attention time on the user interface in Figure 6a, similarly small AOIs in Figure 6b received 35% of attention time and a large and exposed AOIs in Figure 6c received 85% of attention time. The average attention time AOIs received in this scenario is 50%, while the SEQ score for this scenario has a value of 6.16 (see Table 8). 

The results of SEQ scores and gaze fixation durations for AOIs do indicate correlation, as higher SEQ scores are accompanied by a higher percentage of gaze fixation durations for target AOIs. 

Gaze fixations with gaze transitions provide the sequence of the areas to which the individual users paid attention to and can give us additional insight into users’ comprehension of the user interface.

Figure 7 and Figure 8 present gaze fixations and transitions for the “Glavni pohodnik” (English: “Top walker”) Scenario n. 9. The measured time, 25.54 s for User id103 and 22.18 s for User id130 correlate with the visual presentation of gaze fixations, showing that the User id130, needed less time to complete the scenario. Figure 8b shows how User id103 focused first on selection of the appropriate activity type and the correct action (top part of the screen), while User id130 selected the correct action in less time. 

Figure 9 and Figure 10 present gaze fixations with gaze transitions data for Scenario n. 7, where users were required to check the amount of CO_2_ reduction and time of walking activities, in the third week of January. User id130, whose data are presented in Figure 9, needed more time to complete the scenario than User id103, even though the gaze fixations and transitions indicate a different situation. Looking at the comments of the User id130 during evaluation it turns out, that this user reported being confused with the instructions and therefore needed to follow up on that task, which resulted in a longer scenario completion time. At the same time, the user’s gaze was not focused on the screen and resulted in lesser gaze fixations. 

Even though this analysis has been limited to three scenarios, we believe that this approach shows promise in use of these methods as an additional tool for improvement of the user experience and usability evaluations. The eye tracking data alone are of course not sufficient, but can be used as a complementary method for user experience evaluations.

### Method Limitations and Mitigations

One of the limitations is the availability of a suitable inexpensive eye tracking devices and eye tracking software for data collection and analysis. At the time of the study, two devices were suitable; The Eye Tribe Pro and Tobii EyeX. We performed the studies with the Eye Tribe eye tracker, which was subsequently discontinued. The original Eye Tribe Tracker data collection software had only very basic functionality, which we replaced with a more suitable software, namely the Ogama software for eye tracking experiments. High-fidelity mock-ups of the full application were first developed and tested, but the included JavaScript code generated continuous errors and failed tests in Ogama. Therefore, we had to rely on the implementation of static interactive webpages for every single scenario, covering only the functionalities needed to complete the scenarios. This provided some confusion during the study as the users were warned beforehand that some of the buttons will be inactive in a given scenario if they were not needed for its completion. Interviewer in the study paid special attention to this type of problems and encouraged users to try alternative solutions. We did not notice any measurable impact of inactive interactive buttons, except for the fact that a minority of users reported inactive buttons, which in previous scenarios may have been active.

The testing population could have been more diverse in terms of age and demographics, but it still represented typical target users for environmentally oriented applications. A bigger population sample could have provided greater range of users in terms of technical knowledge and experience, but we believe that the present population is still representative.

Concerning the accuracy and reliability of physiological measurements there are several possible error sources. The effects of environment were monitored, e.g., air temperature, lightning conditions that did not interfere with the eye tracker. The same furniture and equipment setup was used during entire study, but the seating position of the users was not static and allowed for same movements of the body. Some users moved out the sensing range of the eye tracker or covered the tracker with hand when clicking on the screen of the tablet.

## 5. Conclusions

The results of our experiment indicate the suitability of an inexpensive eye tracking device for enhancement of user experience evaluations. The collected subjective user data from questionnaires UEQ and SEQ, the experiment observations and objective data measured with the eye tracker device show significant correlation. The attentions maps, areas of interest and gaze fixations and transitions provide additional information related to the user experience and may help with improvements of the user interface after such evaluations. Future work will include testing of other applications, with the goal of user experience improvements, following the ultimate goal of including eye tracking for smart TV applications. 

## Figures and Tables

**Table 1:** The Eye Tribe Pro eye tracking device technical specification.

Sampling Rate	30 Hz to 75 Hz
Accuracy	0.5°–1°
Latency	<1.6 ms
Calibration	6, 9, 12 points
Working range	45–75 cm
Tracking area	45 cm × 30 cm
Screen sizes	Up to 24′′
SDKs	Java, C++ and C#
Data output	Binocular gaze data, head location in 3D
Interface	USB3.0 type C

**Table 2:** Scenario description.

Scenario	Scenario Description
S1	Users should find the application icon on the home screen of the mobile phone, launch the application and proceed to the main screen of the application
S2.	Users should find the “Help” function build in the application and with it explain the main screen elements of the application.
S3	Users should explain the main screen elements of the application without any help.
S4	Users should find the aggregated data for user’s movement, distance covered and CO_2_ savings on the daily basis.
S5	Users should find the aggregated data for distance covered in one month.
S6	User should find the information of his walking distance on Monday.
S7	User should find the information of reduced CO_2_ emissions because of walking in the third week of January.
S8	User should locate and count the number of actions for walking and riding a bike.
S9	User should find action named “Glavni pohodnik” and explain the instructions for its completion.
S10	User should find the screen with gathered general information data.
S11	User should set his own gender in the application settings.
S12	User should turn on the power saving mode.
S13	User should locate his place for running on the scale of participants.

**Table 3:** Gaze fixation duration data for Scenario n. 3—aggregated for all users.

AOI (Index in Figure 4)	Fixation Time (s)	Fixation Percentage
Action (1)	2.15	**3%**
Activity data (2)	189.36	**29%**
Help (3)	24.09	**4%**
Timeline (4)	24.74	**4%**
Selected action (5)	142.74	**22%**
Share (6)	19.21	**3%**
Menu (7)	1.41	**0.2%**

**Table 4:** Gaze fixation duration data for Scenario n. 7—aggregated for all users (for Figure 5c).

AOI (Index in Figure 5c)	Fixation Time (s)	Fixation Percentage
Graph (1)	7.94	15%
CO_2_ selector (2)	4.08	8%

**Table 5:** Gaze fixation duration data for Scenario n. 9—aggregated for all users.

AOI (Index in Figure 6)	Fixation Time (s)	Fixation Percentage
Actions (1)	28.77	30% (of gaze fixation time in Figure 6a)
Walking icon (2)	12.57	16% (of gaze fixation time in Figure 6b)
Action item (3)	15.56	19% (of gaze fixation time in Figure 6b)
Item menu (4)	49.33	49% (of gaze fixation time in Figure 6c)
Item text (5)	36.37	36% (of gaze fixation time in Figure 6c)

**Table 6:** UEQ and SEQ results for two users and scenarios.

User	UEQ Perspicuity	UEQ Efficiency	SEQ Score Scenario n. 7	SEQ Score Scenario n.9	Average SEQ Score
id 103	1.5	1.25	5	5	5.38
id 130	2.75	2.25	6	7	6.38

**Table 7:** Time of completion for Scenarios n. 6, n. 7 and n. 9.

User	Time S6	Time S7	Time S9
id 103	32.03 s	30.19 s	25.54 s
id 130	20.01 s	45.82 s	22.18 s

**Table 8:** Scenario average completion time and SEQ score.

Scenario	Completion (m)	SD	SEQ Score	SEQ SD
S1	20.71	10.88	6.47	0.72
S2	60.79	33.20	6.37	0.87
S3	67.73	24.12	6.59	0.71
S4	38.18	31.34	5.84	1.37
S5	26.42	18.20	6.22	1.10
S6	42.27	31.88	5.74	1.56
S7	53.30	29.69	5.13	1.99
S8	28.07	14.10	6.22	0.91
S9	30.37	19.81	6.16	1.02
S10	24.84	12.61	6.56	0.62
S11	25.42	15.52	6.47	0.67
S12	28.03	13.73	6.50	0.62
S13	51.19	32.35	5.50	1.19
